# Challenges in Pharmacological Intervention in Perilipins (PLINs) to Modulate Lipid Droplet Dynamics in Obesity and Cancer

**DOI:** 10.3390/cancers15154013

**Published:** 2023-08-07

**Authors:** Victória Bombarda-Rocha, Dany Silva, Allal Badr-Eddine, Patrícia Nogueira, Jorge Gonçalves, Paula Fresco

**Affiliations:** 1 Laboratory of Pharmacology, Department of Drug Sciences, Faculty of Pharmacy, University of Porto, 4050-313 Porto, Portugal; up202200055@edu.ff.up.pt (V.B.-R.); up201708266@edu.ff.up.pt (D.S.); allal.badredine@gmail.com (A.B.-E.); up201704254@edu.fc.up.pt (P.N.); pfresco@ff.up.pt (P.F.); 2 UCIBIO–Applied Molecular Biosciences Unit, Associate Laboratory i4HB, Institute for Health and Bioeconomy, Faculty of Pharmacy, University of Porto, 4050-313 Porto, Portugal

**Keywords:** perilipins, lipid droplets, cancer, obesity

## Abstract

**Simple Summary:**

This review highlights the importance of perilipins in lipid metabolism and their potential as therapeutic targets for lipid-associated diseases like cancer and obesity. Perilipins are proteins found in lipid droplets that regulate lipase activity and play a crucial role in maintaining the balance between lipid synthesis and breakdown. Modulating perilipins could improve existing treatments or offer new therapeutic opportunities for addressing these diseases.

**Abstract:**

Perilipins (PLINs) are the most abundant proteins in lipid droplets (LD). These LD-associated proteins are responsible for upgrading LD from inert lipid storage structures to fully functional organelles, fundamentally integrated in the lipid metabolism. There are five distinct perilipins (PLIN1–5), each with specific expression patterns and metabolic activation, but all capable of regulating the activity of lipases on LD. This plurality creates a complex orchestrated mechanism that is directly related to the healthy balance between lipogenesis and lipolysis. Given the essential role of PLINs in the modulation of the lipid metabolism, these proteins can become interesting targets for the treatment of lipid-associated diseases. Since reprogrammed lipid metabolism is a recognized cancer hallmark, and obesity is a known risk factor for cancer and other comorbidities, the modulation of PLINs could either improve existing treatments or create new opportunities for the treatment of these diseases. Even though PLINs have not been, so far, directly considered for pharmacological interventions, there are many established drugs that can modulate PLINs activity. Therefore, the aim of this study is to assess the involvement of PLINs in diseases related to lipid metabolism dysregulation and whether PLINs can be viewed as potential therapeutic targets for cancer and obesity.

## 1. Introduction

In recent years, evidence is being presented to support a major dependence of cancer cells on fatty acid (FA) oxidation to support cell proliferation, survival, stemness, and metastatic progression [1,2,3]. Cancer cells may rely on scavenging the FA extracellular sources of lipids (from neighbor or apoptotic cells) or on endogenous *de novo* lipid synthesis to face their higher dependence on FA oxidation [4]. To prevent lipotoxicity resulting from the presence of free FA in the cytoplasm, cancer cells need to store these FAs efficiently in compartments that can be easily mobilized according to the cell’s needs [2,4,5,6,7,8]; lipid droplets (LDs) are believed to be such compartments [9].

LDs are nano- to micro-sized organelles, composed of a neutral lipid core surrounded by a phospholipid monolayer [10,11]. For a long time, LDs were considered inert lipid storage organelles but, after the discovery of proteins anchored to their surfaces [12], they became recognized as functional organelles, with key roles in lipid metabolism, energy homeostasis, and communication between distinct cellular signaling pathways [11,13,14].

Perilipins (PLINs), the most abundant proteins in LDs, regulate LD stability and lipid turnover, consequently controlling the overall lipid metabolism [15]. Given the involvement of lipid droplets in cancer pathophysiology [9], understanding the roles of perilipins could help clarify how changes in the lipid metabolism affect cancer cells and shed light on the potential impact of these metabolic alterations on cancer progression. The goal of this study is to examine the primary roles of perilipins, especially in cancer, with the aim of exploring their potential as therapeutic targets for cancer treatment. PLINs play a role in maintaining the equilibrium among various FA stores within the body and contribute to the onset of conditions like obesity and irregular fat accumulation in non-adipose tissues, as seen in liver steatosis and atheroma plaque formation [16]. Given that obesity is a known risk factor for cancer, the link between conditions predisposing to obesity and cancer will also be explored.

## 2. LD Biogenesis

**Figure 1:**
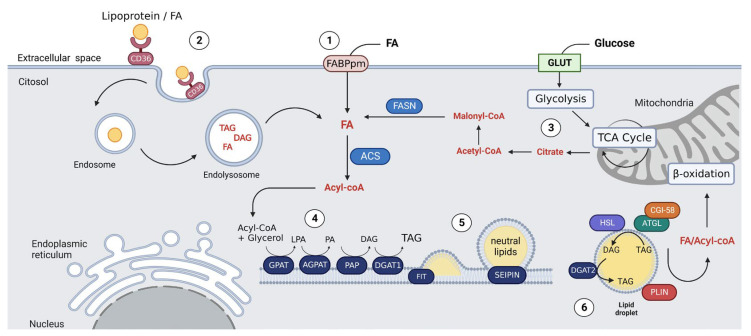
Overview of LD biogenesis in cancer cells. The figure illustrates the possible sources of FAs and the mechanisms involved in LD biogenesis: Free FA uptake can occur through FABPpm (1) and, when combined with lipoproteins, uptake occurs through CD36-mediated internalization, through a clathrin-mediated endocytosis (2). These FA scavenging mechanisms may be complemented by FA *de novo* synthesis (3). The FAs absorbed/synthetized will be esterified into neutral lipids and can be transported to the ER or to LD. TAG synthesis starts with the activation of FA into Acyl-CoA and follows in the ER by action of the esterification enzymes (4). The newly formed neutral lipids accumulate between the ER bilayers, where LD biogenesis occurs. SEIPIN stabilizes the LD structure while FIT proteins help in the portioning of neutral lipids (5). Once fully formed, the LD will be released into the cytosol, carrying a set of proteins and enzymes for managing the lipid cargo in response to lipolytic stimuli (6). Abbreviations: ACS (acyl-CoA synthetase); AGPAT (acylglycerol-P acyltransferase); ATGL (adipose triacylglyceride lipase); CGI-58 (comparative gene identification 58 protein); DAG (diacylglyceride); DGAT (diacylglycerol acyltransferase); FABPpm (plasma membrane fatty acid-binding proteins); FA (fatty acid); FASN (fatty acid synthase); FIT (fat-storage inducing transmembrane); GLUT (glucose transporter); GPAT (glycerol-P acyltransferase); HSL (hormone sensitive lipase); LPA (lysophosphatidic acid); PA (phosphatidic acid); PAP (phosphatidic acid phosphohydrolase); PLIN (perilipin); TAG (triacylglyceride); TCA cycle (tricarboxylic acid cycle).

The biogenesis of LDs mainly takes place within the endoplasmic reticulum (ER), culminating in the creation of a fully functional structure [10]. A schematic representation of this process is depicted in Figure 1.

The LD structure comprises a core of neutral lipids enveloped by a single layer of phospholipids with proteins [10]. The core of neutral lipids is composed of esterified cholesterol or FAs, which can be obtained either through external uptake or via *de novo* synthesis [17].

The uptake of FA occurs through transporters such as CD36, the fatty acid transport protein family (FATP), and plasma membrane fatty acid-binding proteins (FABPpm) [18]. CD36 is recognized as a multifunctional membrane scavenger receptor [18]. When free FA binds to CD36, the resulting complex is internalized, forming an endosome. This endosome then transports the FA into the cell, possibly directly to the LD, where it is esterified and stored as triacylglycerol (TAG) [19]. FABPs function as enzymes, being able to convert FA into FA-acyl-CoA. The FA-acyl-CoA formed can translocate to the inner side of the membrane (aqueous phase) and bind to the cytoplasmic fatty acid-binding protein (FABPc) [20], which will shuttle FAs to different cellular compartments [21].

*De novo* lipogenesis, the process of producing new FAs, originates from citrate and acetate, which are produced by pyruvate oxidation via the TCA cycle [22]. This process primarily occurs in hepatocytes and adipocytes under normal physiological conditions [22]. However, this process is reactivated in cancer cells, seemingly as an adaptive strategy to produce large amounts of FA, which can then be lengthened or desaturated for various critical functions of cancer cells survival [22].

Within the ER, FAs are transformed into neutral lipids, specifically in the form of TAG [23]. The synthesis of TAG starts with the activation of FA through the addition of acyl-CoA, a reaction catalyzed by the enzyme acyl-CoA synthetase (ACS) [23]. This reaction produces FA-acyl-CoA, which is then esterified with glycerol-3-phosphate by the enzyme glycerol-P acyltransferase (GPAT), resulting in the formation of lysophosphatidic acid (LPA) [23]. LPA acts as a substrate for acylglycerol-P acyltransferase (AGPAT), which catalyzes the addition of another FA-acyl-CoA, yielding phosphatidic acid (PA) [23]. PA is then used by phosphatidic acid phosphohydrolase (PAP) to create diacylglycerol (DAG), which is finally esterified into TAG by the enzyme diacylglycerol acyltransferase (DGAT) [23]. DGAT, the enzyme responsible for the final step of TAG synthesis, has two isoforms, DGAT1 and DGAT2 [24]. DGAT2, found in LD membranes, handles the esterification of FA within the LD [24]. This isoform forms a complex with another enzyme, monoacylglycerol acyltransferase 2 (MGAT2), which is instrumental in converting monoacylglycerol into DAG [24].

The TAG synthesized by DGAT starts to accumulate between the bilayer membrane of the ER, marking the beginning of LD biogenesis [11,25]. Continuous accumulation of TAG builds lens-like structures which eventually break away, or bud, from the ER [11,25]. This budding process is driven by the interfacial tensions between the LD and the cytosol [25]. This stage requires the involvement of additional proteins that facilitate the expansion of the neutral lipid core and help stabilize the TAG aggregates [25]. The proteins involved in this process primarily consist of fat storage-inducing transmembrane proteins (FIT or FITM) and SEIPIN [26,27]. FIT proteins are localized to the ER, and both of their isoforms, FIT1 and FIT2, participate in LD biogenesis [26,27]. Rather than synthesizing neutral lipids, FIT1 and FIT2 assist in integrating TAG into LDs [26,27]. When FIT proteins are overexpressed, they result in LDs enriched with TAG [26]. SEIPIN, also known as Berardinelli–Seip congenital lipodystrophy type 2 or BSCL2 protein, is a transmembrane protein in the ER and it plays a pivotal role in shaping LD [28]. It is suggested that SEIPIN stabilizes the membrane bridges between the ER and LD, acting as a contact point and enabling the transfer of neutral lipids into LD [29,30]. In the absence of SEIPIN, the budding process is delayed and becomes heterogeneous [31]. In humans, deficiency in SEIPIN can cause lipodystrophy (an abnormal distribution and/or partial loss of adipose tissue), neurological defects, and multi-organ problems [31].

A group of other proteins, the PLINs, also participate in the regulation of the rate of lipolysis in the LD [32]. These proteins are transferred from the Golgi to the ER via the coat complex protein I (COPI) and ADP-ribosylation factor 1 (ARF1) machinery [33]. When LDs complete their formation, they detach from the ER already fully functional and able to toggle between lipogenic and lipolytic pathways as per the cell’s needs [34].

The balance between lipogenesis and lipolysis is directly influenced by the accessibility of lipases to the lipid core of the LD [11]. PLINs play a crucial role in regulating this accessibility and thus are key regulators of lipid metabolism [32].

## 3. PLINs as LD Gatekeepers

PLINs, also known as PAT proteins, represent the most prevalent proteins found in LD membranes [32]. Five PLINs have been identified in mammals (PLINs 1–5) and all serve as crucial proteins for the stabilization and protection of LDs, responding to either lipogenic or lipolytic stimuli [35]. They function by preventing lipases from accessing the content of LDs [32,35,36,37]. The regulation of PLINs’ efficacy or activity is influenced by both the expression levels of individual variants and the activity of kinases [32]. The latter is dependent on the metabolic state of the cell or the energy requirements of the organism [32]. Given that each PLIN has a unique expression pattern [38], the distribution of lipids throughout the human body is determined by the collective contribution of each PLIN, in accordance with its specific control mechanisms and tissue distribution. To deepen the role of perilipins in systemic lipid distribution, a more comprehensive overview of each perilipin is provided as follows.

### 3.1. Perilipin 1

PLIN1 is mainly found in mature adipocytes, where it acts as a lipolysis modulator, thereby regulating lipid redistribution from adipose tissues to other organs and tissues [39]. PLIN1 regulates the access of the lipases to the LD content [39]. PLIN1 is bonded to comparative gene identification-58 (CGI-58), a co-activation factor of adipose triglyceride lipase (ATGL), preventing ATGL activation and lipase access to stored TAG [39]. The activity of CGI-58 depends on the energy state of the organism. When in the fed state, CGI-58 remains combined with PLIN1 [39,40]. Conversely, after prolonged fasting, CGI-58 is released from PLIN1, binds with ATGL, and activates ATGL lipase activity [39,40].

**Figure 2:**
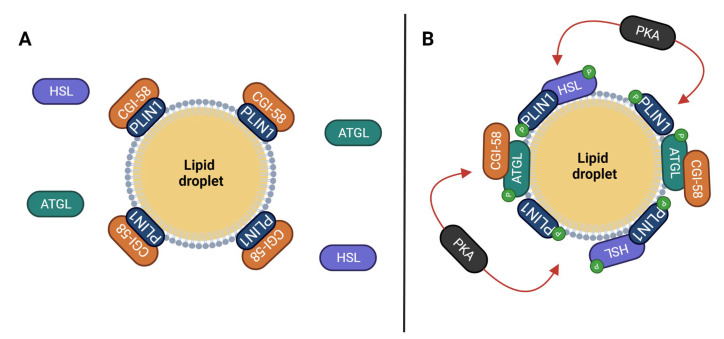
Influence of PLIN1 activation on the assembly of lipolytic enzymes in the lipid droplet (LD). Panel (**A**): In basal state, CGI-58 remains attached to PLIN1 avoiding interaction with ATGL and consequent co-activation of the lipase. The lipid content of the LD stays protected from lipolytic activity. Panel (**B**): In stimulated state, PKA phosphorylates PLIN1 and the interaction with CGI-58 is broken. CGI-58 binds to phosphorylated ATGL, and the lipase is fully activated. Phosphorylated HSL binds to phosphorylated PLIN1, which allows access to the lipid content of the LD. Abbreviations: ATGL (adipose triacylglyceride lipase); CGI-58 (comparative gene identification 58 protein); HSL (hormone sensitive lipase); P (phosphate); PKA (protein kinase A); PLIN1 (perilipin 1).

The lipolytic pathway of PLIN1 is controlled by receptors linked to the cAMP-dependent protein kinase (PKA) pathway [39]. Phosphorylation of PLIN1 induced by PKA disrupts the bond between PLIN1 and CGI-58, setting free CGI-58 to bind to ATGL; PKA also phosphorylates ATGL, thereby increasing its affinity for free CGI-58 [40]. Additionally, PKA phosphorylation uncovers the hormone-sensitive lipase (HSL) binding site in PLIN1, which allows the lipase to access the diacylglycerol (DAG) and monoacylglycerol (MAG) within the LDs [41,42]. Figure 2 shows a schematic representation of PLIN1 in basal and stimulated states.

PLIN1 is an important regulator of lipolysis in white adipose tissue (WAT) and it is associated with both basal and adrenergic-induced lipolysis [43]. The PLIN1 gene locus has been associated with variability in weight loss [44]. Polymorphisms [45], epigenetic modifications [46], and varying levels of PLIN1 expression [47,48,49] have also been associated with obesity phenotypes and with the success rate of weight loss following bariatric surgeries [50].

In cancer, PLIN1 overexpression has been reported to favor cancer development in tissues which normally express PLIN1 (adipose tissue) [51], being highly expressed in sebaceous adenomas and carcinomas [52] and proposed to be a potential biomarker for liposarcomas [53]. PLIN1 overexpression was also found in hepatic tumors, including hepatocellular adenoma and carcinoma [52]. In breast cancer cells, PLIN1 expression was shown to prevent cancer cell evasion and tumorigenesis and reduced PLIN1 expression was correlated with a poorer prognosis in breast cancer patients [54,55]. These studies seem to indicate the distinct role of PLIN1 in cancer according to the FA management profile of each cell type. In cells that function as FA storage cells (adipocytes and, to some extent, hepatocytes), PLIN1 overexpression may favor tumorigenesis whereas in cells that are end-users of FA (like cancer cells), a poor expression of PLIN1 may limit the cell’s ability to store FA and, hypothetically, force the cell’s evasion to find a place more favorable to have access to energy.

### 3.2. Perilipin 2

PLIN2, also known as adipophilin, is ubiquitous and continuously expressed [56,57,58]. PLIN2 cell levels are controlled by its rates of synthesis and degradation [56]. PLIN2 binds to the LD membrane and acts as a barrier to lipolysis by preventing the access of the lipases to the lipids stored in the LD, meaning the LD permeability to lipolytic enzymes is proportional to the cellular PLIN2 [32,58,59,60].

Expression of PLIN2 is induced by peroxisome proliferator-activated receptor gamma (PPARγ) activation [61,62,63]. PPARγ activators and high fat diets were reported to increase PLIN2 expression [61,63,64,65,66,67]. PPARγ is predominantly expressed in adipose tissue [68,69]. Therefore, these stimuli may promote the retention of TAG inside the LDs of adipocytes by a PPARγ-induced increase in PLIN2 expression.

The ubiquitin–proteasome pathway consistently degrades expressed PLIN2, a process that involves the E3 ubiquitin ligase (Ubr1) [70,71]. Ubr1 targets PLIN2 for degradation in an amino acid-dependent manner [71]. This increase in Ubr1 activity results in enhanced ubiquitination of PLIN2, which facilitates the access of lipolytic enzymes to the LD core. In turn, this leads to an elevated bioavailability of FA for β-oxidation, thereby fueling the increase in the cell’s anabolic activity triggered by the availability of amino acids [72]. Reciprocally, PLIN2 overexpression was shown to refrain protein synthesis translated in decreased muscle thickness and strength [72].

**Figure 3:**
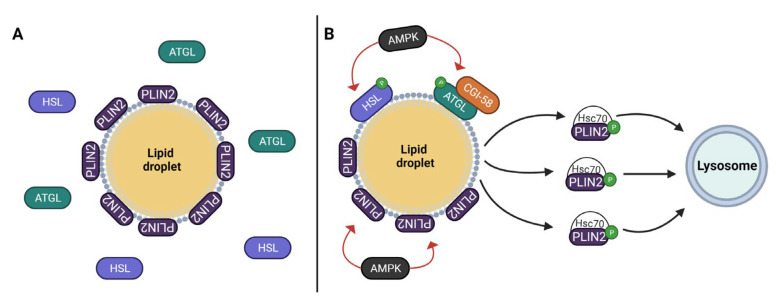
Influence of PLIN2 activation on the assembly and access of lipolytic enzymes in the lipid droplet (LD). Panel (**A**): In basal state, PLIN2 stays attached to the LD surface, protecting the LD content from the lipase activity as a barrier. Panel (**B**): In stimulated state, AMPK phosphorylates PLIN2. Phosphorylated PLIN2 binds to Hsc70 and is subsequently carried to the lysosome for chaperone-mediated autophagy degradation. Without PLIN2, the LD is vulnerable to the lipolytic activity of the lipases. Abbreviations: AMPK (AMP-activated protein kinase); ATGL (adipose triacylglyceride lipase); CGI-58 (comparative gene identification 58 protein); Hsc70 (heat shock cognate 70 kDa protein); HSL (hormone sensitive lipase); P (phosphate); PLIN2 (perilipin 2).

PLIN2 bound to the LD membrane is degraded by chaperone-mediated autophagy (CMA), as illustrated in Figure 3 [73,74]. CMA involves a coordinate action of heat-shock cognate protein of 70 kDa (Hsc70) and lysosome-associated membrane protein type 2A (LAMP-2A), which are also present in LDs [75]. CMA of PLIN2 starts with the binding of PLIN2 to Hsc70, which interacts thereafter with LAMP-2A, culminating with PLIN2 degradation [76].

CMA of PLIN2 may be increased by its phosphorylation by AMP-activated protein kinase (AMPK) [74]. Under conditions of starvation, specifically when glucose is low, ATP concentrations will drop, increasing the AMP/ATP ratio [74]. This elevated ratio is detected by starvation sensors like AMPK, which promote CMA of PLIN2 [74]. This degradation allows lipolytic enzymes access to the content of LDs, resulting in the mobilization of FA as alternative energy sources to counteract starvation [74,77]. However, the response to an increase in AMP/ATP ratio seems to be context-dependent, as is demonstrated by the alterations triggered by hypoxia.

Hypoxia induces an extensive metabolic reprogramming of the cell to face the decrease in nutrients and oxygen supply. The metabolic reprogramming is coordinated by the hypoxia-inducible factors (HIF) 1-alpha (HIF-1α) and 2-alpha (HIF-2α) [78]. Under normoxic conditions, HIF-1α and HIF-2α are continuously ubiquitinated for proteasomal degradation [79,80]. Hypoxia prevents HIFs degradation and allows their translocation to the nucleus, where they combine with HIF-1β altering gene transcription so the cell can endure hypoxic conditions [78]. The PLIN2 gene, also known as ADRP (Adipose Differentiation-Related Protein) gene, is one of the genes induced by HIF-1α and by HIF-2α [81,82]. Additionally, HIF-1α also induces the expression of hypoxia-inducible lipid droplet-associated (*hilpda)*, the gene that encodes the hypoxia-inducible protein 2 (HIG-2) [83] whose function is the inhibition of ATGL-mediated lipolysis [84,85]. Hypoxia ends up promoting an increase in lipid storage in LDs, by increasing PLIN2 and inhibiting the activity of lipolytic enzymes.

The interplay between PLIN2 and hypoxia seems to be particularly relevant as PLIN2 has been shown to be involved in a positive loop that further inhibits the degradation of HIF-1α and sustains its expression and that of *hilpda* and, therefore, the increase in LD load promoted by hypoxia [86]. The pathophysiological consequences of this loop may be vast and are far from being totally understood as hypoxia or “pseudo-hypoxic conditions” (i.e., conditions that prevent HIF-1α and HIF-2α degradation even in the presence of oxygen), and may be relevant in promoting obesity [87,88] or cancer [89]. The association between PLIN2 and cancer is extensively documented. PLIN2 has been associated with tumorigenesis and is often considered as a poor prognosis indicator in cancers of the colon [90,91], breast [92,93], prostate [94,95], lung [96,97], bladder [95], kidney [82,98,99,100,101,102,103,104,105], thyroid [106], gastric [107], and melanoma [108].

PLIN2 was also shown to be involved in the interaction between LDs and the plasma membrane [109,110,111], and to play a pivotal role in both the formation and secretion of milk lipids [112]. Therefore, PLIN2 may also participate in intercellular signaling. PLIN2 was shown to act as an adipokine [113,114,115], promoting the activation of the inflammatory and fibrotic processes of progressive liver injury [116], the development of atherosclerotic arterial plaques in the cardiovascular system [117], and alterations of the phenotype of microglia and of other macrophages towards a more inflammatory phenotype in the brain [118]. The alterations in the phenotype of brain inflammatory cells induced by PLIN2 have been related to neuroinflammation and oxidative stress [118], neurodegeneration [119], the suppression of remyelination [120,121], cognitive impairment [122] and accelerated aging [119,123].

### 3.3. Perilipin 3

PLIN3, also known as TIP47, is ubiquitously expressed [124]. During the nucleation phase of LD biogenesis, PLIN3 is mobilized to the nascent LD [35,125]. Over time, PLIN3 may be progressively replaced by PLIN2 [35,126]. Functionally, PLIN3 behaves much like PLIN2, restricting lipases’ access to the LD core. Its degradation is also mediated by CMA and regulated by AMPK [73].

PLIN3 seems to play a pivotal role in promoting the transition from brown to WAT and, consequently, in obesity. The depletion of PLIN3 in WAT triggers the generation of brown adipocytes and the expression of genes responsible for thermogenesis [127].

PLIN3 also appears to play a significant role in cancer pathogenesis. Studies have reported abnormal PLIN3 expression in several types of cancer, including cervical [128], clear cell renal cell [129], breast [93], lung [130], and prostate [131,132,133]. The prevalence of high PLIN3 levels in these cancer types could suggest an increased rate of LD biogenesis or potentially hindered conditions for PLIN3′s CMA. It is noteworthy that the upregulation of PLIN3 expression has been observed to result in decreased efficacy of enzalutamide treatment and radiotherapy (thus increasing resistance) [131,133]. On the contrary, it has been demonstrated that PLIN3 can enhance the therapeutic efficacy of docetaxel by reducing resistance to therapy [132]. These seemingly contradictory effects might shed light on intricate aspects of cancer metabolism, underscoring the proposed significance of LD in both cellular metabolism (availability of cholesterol for androgen synthesis and of FA to compensate blockade of endocytic pathways) and in stress management (protecting the cells from the stress induced by the lipid peroxidation caused by radiotherapy) [134].

### 3.4. Perilipin 4

PLIN4, also known as S3-12, is the least studied protein of the perilipin family [135]. It is mostly identified in preadipocytes and in membranes of nascent LDs [135,136]. It is suggested that PLIN4 is involved with adipocyte differentiation and, to promote stability of LD membrane, acts as a surfactant [59]. PLIN4 location has also been correlated to cholesterol ester rich LDs [137,138] and its expression is also induced by PPARγ activation [61].

The depletion of PLIN4 has been linked to diminished PLIN5 expression in the heart and a reduction in heart LDs. This suggests that the functions of PLIN4 and PLIN5 may be closely interconnected [139].

The most solid evidence pointing to a relationship between PLIN4 and obesity was provided by the observation that single nucleotide polymorphisms of PLIN4 were correlated with obese phenotypes [140]. In cancer, PLIN4 is highly expressed in luminal A and B breast carcinomas [93] and its expression is associated with triple-negative breast cancer resistance to cytotoxic chemotherapy [141].

### 3.5. Perilipin 5

PLIN5, also known as OXPAT, predominantly occurs in tissues that rely on β-oxidation for energy generation, such as skeletal muscle, cardiac muscle, and brown adipose tissue [142]. Its expression has also been documented in the epithelial cells of the gastrointestinal and urogenital tracts, hepatocytes, renal tubular cells, ductal cells of the salivary glands, and pancreatic cells [143].

**Figure 4:**
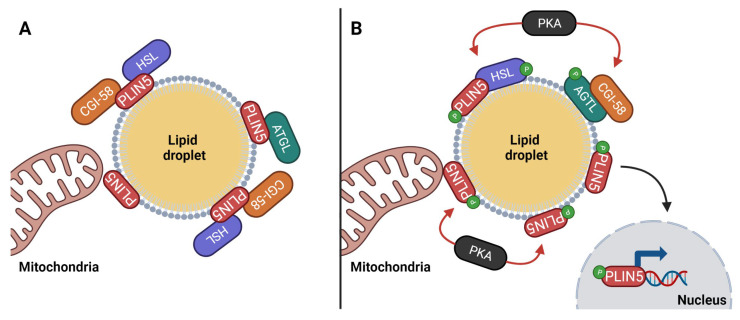
Influence of PLIN5 activation on the assembly and access of lipolytic enzymes in the lipid droplet (LD). Panel (**A**): In basal state, PLIN5 binds to CGI-58 and ATGL, preventing their interaction and consequent lipolytic activity. Panel (**B**): In stimulated state, PKA phosphorylates PLIN5 and its interaction with CGI-58 and ATGL is undone. CGI-58 binds to phosphorylated ATGL and the lipase is fully activated. Phosphorylated HSL is active and it keeps bonded to phosphorylated PLIN5, which allows access to the lipid content of the LD. Phosphorylated PLIN5 can also travel to the nucleus, where it binds to sirtuin1 and peroxisome proliferator-activated receptor gamma coactivator 1-alpha to activate the transcription of genes related FA catabolism, mitochondrial biogenesis, and respiration. PLIN5 is also known to be a contact site between LDs and mitochondria during β-oxidation. Abbreviations: ATGL (adipose triacylglyceride lipase); CGI-58 (comparative gene identification 58 protein); HSL (hormone sensitive lipase); P (phosphate); PKA (protein kinase A); PLIN5 (perilipin 5).

PLIN5 is found at the contact site between LDs and mitochondria [32,37]. The relevance of such location raises the question of whether LD–mitochondria contact sites exist to promote the transfer of FAs from LDs to mitochondria, or to shield the cell from an overload of FAs that the mitochondria cannot process [32,37]. Findings that PLIN5 overexpression induces cardiac steatosis, and that PLIN5 ablation reduces cardiac LD formation [144], suggest that PLIN5 plays a role in a cellular adaptive response to high lipid oxidative metabolism [144,145,146,147]. These observations that support the protective role of PLIN5 from excessive FA load are further supported by the observation that PLIN5 is regulated by AMPK and that AMPK activation increases PLIN5 expression and LD formation, and mitigates cellular oxidative stress by lowering the levels of reactive oxygen species in hepatic stellate cells [146]. However, PLIN5 has also been shown to interact with both CGI-58 and ATGL, inhibiting the lipolytic activity of ATGL through a dual mechanism regulated by PKA [148,149], as illustrated in Figure 4. Any stimuli that result in PKA activation—such as cold exposure, physical activity, fasting, or other stress factors—will induce PLIN5 phosphorylation. PLIN5 phosphorylation leads to its detachment from CGI-58 and ATGL, subsequently activating ATGL’s lipolytic activity [144]. Besides its role in regulating access to LD reserves, PLIN5, when phosphorylated by PKA, translocate to the nucleus. There, it interacts with sirtuin 1 (SIRT1), activates peroxisome proliferator-activated receptor gamma coactivator 1α (PGC-1α), and stimulates the transcription of genes of FA catabolism, mitochondrial biogenesis, and respiration [144]. Through this PKA-mediated regulation, PLIN5 not only facilitates FA oxidation but also increases the cell’s FA oxidative capacity [144,149].

Therefore, the function of PLIN5 seems to participate in a delicate balance to preserve lipid homeostasis [149]. It favors β-oxidation metabolism by allowing the release of FA and promoting the synthesis of the required enzymatic machinery when the cell needs to meet a higher energy demand [149]. Yet, it is also capable of preventing excessive lipolysis, shielding the cell against lipotoxicity when the concentration of FAs in the cytoplasm exceeds the amount that mitochondria can handle, or when signs of oxidative stress are present [149].

In the context of cancer, the overexpression of PLIN5 has been observed in patients with breast cancer, renal carcinomas, liposarcoma, rhabdomyosarcoma, and leiomyosarcoma [93,143]. It is also significantly prevalent in hepatocellular carcinomas [150], where it serves as a biomarker linked to unfavorable prognosis for patients suffering from this type of liver cancer, and it has been associated with the evolution of non-alcoholic fatty liver disease to liver cancer [151].

## 4. Coordination of PLINs’ Function

The diverse roles and tissue distribution of PLINs raise the question about how their actions are coordinated to act both as gatekeepers of LD and to allow the release of LD contents when required [32]. The orchestration of PLINs’ function can be achieved through the concerted action systems that monitor the availability of energy and nutrients for metabolic processes [38].

Peroxisome proliferator-activated receptors (PPARs) are transcriptional factors that control the expression of genes that code for proteins involved in FA storage, glucose metabolism, and adipocyte differentiation [152], including PLINs, as described above [142,153,154]. Out of the three known PPAR isoforms (α, β/δ, and γ), PPARγ and PPARα have the most significant impact on the function of PLINs. PPARγ is ubiquitously expressed but the tissue distribution can vary among its three mRNA splice variants: PPARγ1, PPARγ2, and PPAγ3. PPARγ1 is also ubiquitously expressed, while PPARγ2 is predominantly found in adipose tissues and the liver and PPARγ3 is mainly expressed in the colon and adipose tissue [155]. On the other hand, PPARα is primarily expressed in skeletal muscle, brown adipose tissue, and the liver, playing a key role in the regulation of β-oxidation [156].

PPARγ activity can be influenced by substances like polyunsaturated FAs, eicosanoids, and oxidized lipid components [157]. Elevated levels of FAs activate PPARγ, which in turn boosts the loading capacity of LDs. This boost operates through a dual mechanism: first, by promoting adipocyte differentiation, which increases the number of cells capable of storing FAs in LDs and, second, by directly enhancing LD loading [158].

Two specific mechanisms seem to underpin the enhancement of LD loading mediated by PPARγ. One is the upregulation of PLIN1 expression, which improves the capacity to restrict lipolytic enzymes from acting primarily on LDs in adipocytes [153]. The other involves an increase in PLIN2 expression, which bolsters the ability to block lipolytic enzymes’ access to the LD core [61,63,64,65,66,67].

In addition to the influence on PPARγ, free FAs also stimulate PPARα, resulting in increased β-oxidation in skeletal muscle and the liver [156,159]. Thus, when free FA levels rise, PLINs in adipose tissue, liver, and other target organs coordinate their activities to promote a balanced redistribution of FAs between the adipose tissue and the target organs. This coordination allows these tissues to expand their capacity to store more FAs within LDs and to utilize more FAs for β-oxidation.

Any stimulus that activates PKA can lead to PLIN1 phosphorylation by PKA [39]. PLIN1 phosphorylation inhibits its ability to restrict the access of lipolytic enzymes to the LD’s core in adipocytes, which in turn increases the levels of FAs in the plasma [39]. These FAs can activate PPARs [159], triggering a process like the one described above.

The key distinction between PKA-induced regulation and PPARγ-induced regulation lies in the effect on adipocytes’ ability to store FAs in LDs. Under PKA regulation, this ability is diminished due to PKA’s action on PLIN1 [39]. This results in an imbalanced distribution of FAs, causing a preferential flow from the adipose tissue to the target organs. Furthermore, PKA modulates PLIN5, which also facilitates the transfer of FAs to mitochondria. This not only enhances the use of FAs as energy substrates but also creates an FA gradient to favor the mobilization of FAs from adipose tissues.

In situations where a cell has the necessary conditions for proliferation (such as the availability of sufficient amino acids), this mechanism may also be triggered. By sensing the abundance of these anabolic components, the cell promotes PLIN2 degradation as well [72]. This process increases the available energy sources to meet the heightened demands imposed by anabolic activity [72].

This coordinated regulation can fail if either the sensitivity of PLIN1 to phosphorylation decreases or the control over PLIN2 degradation lessens. In such cases, obesity or ectopic fat deposits may occur, leading to dysfunctions in lipid metabolism and the potential development of associated diseases [116]. In fact, studies have shown that the absence of PLIN2 in mice fed a high-fat diet (HFD) prevented HFD-induced obesity [116]. This outcome was associated with an increase in the formation of subcutaneous beige adipocytes expressing uncoupling protein 1 (UCP-1), as well as reduced formation of inflammatory foci in WAT and the reduction of liver steatosis [116]. Moreover, it was also observed that a loss of PLIN2 resulted in reduced energy intake and increased physical activity in mice subjected to HFD feeding [116].

In the context of cancer, the overexpression of PLIN2 and PLIN3 is often observed [90,91,92,93,95,96,99,101,105,106,107,108,128,129,130,131,132,133,160,161,162,163,164]. Considering their individual roles, this suggests that cancer cells might possess an enhanced ability to generate new LDs, as PLIN3 typically associates with nascent lipid droplets, and a heightened capability for lipid storage, since overexpression of PLIN2 could make lipid droplets less accessible to lipolytic enzymes [35]. Therefore, the overexpression of PLIN3 and PLIN2 in cancer cells could extend beyond an augmented generation of lipid droplets, as it may also denote heightened efficacy in lipid preservation. This could provide a competitive advantage over ‘normal’ cells that do not overexpress PLIN3 and PLIN2, such as adipocytes.

Several prevalent types of cancer, such as breast, colorectal, esophageal, kidney, gallbladder, uterine, pancreatic, and liver, have been associated with obesity, which also increases the likelihood of cancer mortality [165,166]. The population-attributable fraction, a measure of the burden of cancer attributable to obesity, is 11.9% for men and 13.1% for women [165]. Proximity to adipose tissue has been observed to favor the growth or development of metastasis in many epithelial tumors [167,168].

Adipocytes and cancer cells appear to establish a metabolic symbiosis, transforming adipocytes into cancer-associated adipocytes and promoting cancer growth [167]. This transformation involves the mobilization of FAs from adipocytes, a process that contributes to tumor progression [168]. Various factors likely play a role in this mobilization, including alterations in the expression and activity of PLINs. The coordination of PLINs by both PPARs and PKA pathways may be particularly relevant.

Adrenaline is the endogenous ligand of β_2_-adrenoceptors, which are prototypically coupled to the Gs/cAMP/PKA pathway [169]. Both our own research [170,171] and that of others [172,173] have demonstrated that tumorigenic cancer cells have the capacity to synthesize and release adrenaline, creating conditions for adrenergic activation in the tumor microenvironment independent of sympathetic or hormonal stimulation. Therefore, the presence of this PKA regulation, which enables the release of adipocyte LD control by PLIN1, combined with the increased capacity of PLIN2 to retain FAs in the LDs of cancer cells via the FA/PPARγ pathway, shifts the adipocyte/cancer cell balance in favor of FA retention by cancer cells.

Ultimately, the accumulation of FAs in the LDs of cancer cells, along with their ability to mobilize FAs from adipocytes in the tumor microenvironment, may enhance the ability of cancer cells to resist the starvation conditions signaled by AMPK activation [2].

## 5. Pharmacological Interventions

The significance of PLINs in regulating lipid metabolism is undeniable. However, they have not yet been prioritized as targets for pharmacological intervention. Nevertheless, there are a variety of drug categories, some of which are approved for clinical use, that operate on the regulators of PLIN function or expression. This repertoire includes PPAR activators [174], SIRT1 [175,176], and AMPK activators [177], as well as drugs that interact with receptors linked to the Gs/cAMP/PKA pathway [178].

**Figure 5:**
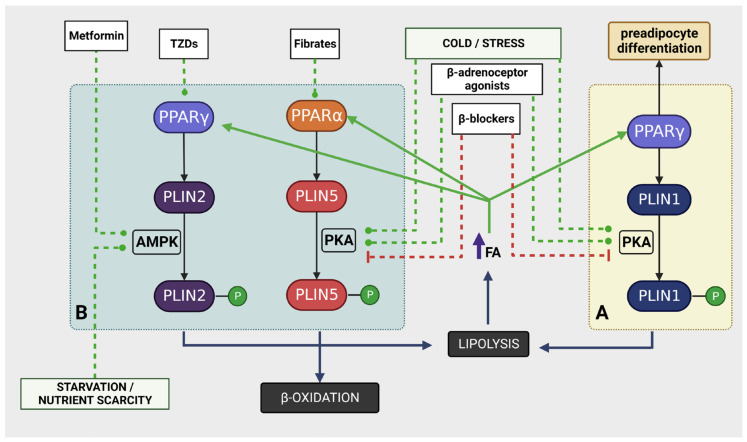
PLIN’s coordination of FA storage and possible pharmacological intervention strategies. Increase in the body’s need for FAs for β-oxidation is signaled through hormone-dependent PKA activation, which leads to PKA-mediated lipolysis and increase of β-oxidation to fulfill the body’s energy demands. This mechanism is partly orchestrated by the type of PLIN expressed in each cell type. In adipocytes (Box A), PKA induces PLIN1 phosphorylation, allowing lipolytic action over the lipid droplet (LD) lipid content. In β-oxidative cells (Box B), PKA induces PLIN5 phosphorylation, favoring FA transfer to mitochondria and β-oxidation. Conditions of starvation/nutrient scarcity (low ATP/AMP ratio) are signaled through AMPK. AMPK activation induces PLIN2 phosphorylation, allowing the lipolytic action over the LD lipid content (see text for details). The increase in free FA availability will stimulate PPARs. PPARγ activation in adipocytes will stimulate adipogenesis (preadipocyte differentiation) and expression of PLIN1 and PLIN2, increasing the storage capacity and their capacity to react to hormone-induced FA mobilization. PPARγ activation in β-oxidative cells will increase mainly PLIN2 expression, favoring FA storage in LDs and the capacity of these cells to react to local starvation conditions. Increased FA availability will also activate PPARα, which stimulates PLIN5 expression in β-oxidative cells, increasing their β-oxidative capacity to react to hormone stimulation. Pharmacologically, PPARs, PKA, and AMPK can be modulated by widely used drugs such as TZDs (PPARγ agonists), fibrates (PPARα agonists), metformin (AMPK activator), and adrenoceptor agonists and antagonists (modulation of PKA pathway). In the figure, green lines indicate activation and red lines indicate inhibition. Abbreviations: AMPK (AMP-activated protein kinase); FA (fatty acid); LD (lipid droplet); P (phosphate); PKA (protein kinase A); PLIN1 (perilipin 1); PLIN2 (perilipin 2); PLIN5 (perilipin 5); PPARα (peroxisome proliferator-activated receptor alpha); PPARγ (peroxisome proliferator-activated receptor gamma); TZDs (thiazolidinediones).

These regulatory pathways exert unique influences on different PLINs [41,142,146,149,153,154]. Therefore, their impact on the coordination of PLIN functions and on the allocation of FAs between adipocytes and recipient cells may also be distinct. A comparative analysis of these different scenarios will be undertaken, juxtaposing theoretical expectations with empirical data concerning the established effects of these drugs on obesity and cancer risk. Figure 5 illustrates the coordination of perilipins, with a focus on PLINs 1, 2 and 5, and possible pharmacological interventions.

### 5.1. PPAR Activators

As previously described, PPARs play a significant role in maintaining nutrient homeostasis and they are the main regulators of PLIN expression [142,153,154]. Therefore, the pharmacological modulation of PPAR activity is directly associated with the modulation of the energy metabolism [174,179]. Even though this effect is already vastly explored in clinical practice for the treatment of hyperglycemia and dyslipidemias, it is also a target of interest for metabolic diseases such as obesity and cancer [180].

Fibrates are high affinity PPARα activators used for the treatment of dyslipidemias [181,182]. Through the activation of PPARα, these drugs enhance the cellular uptake of FA and their breakdown through the β-oxidation pathways [182]. Besides activating other genes related to peroxisomal and mitochondrial FA oxidation, PPARα induces PLIN5 expression [183], which can explain the weight lowering effect of fibrates, since PLIN5 is involved in the PKA-mediated lipolytic mechanisms and β-oxidation [149]. The prioritization of the oxidation of FAs over glycolysis and the disturbance of the equilibrium between glucose and lipid metabolism are a possible explanation of the potential anti-tumorigenic activity of PPARα agonists in cancer treatment [184].

Thiazolidinediones (TZDs), recognized as PPARγ activators, are utilized in the management of diabetes [185]. Considering the established tendency of PPARγ activators to induce adipogenesis [186] and upregulate PLIN2 [61,62,63] expression in non-adipose cells, it is plausible that these medications might foster fat accumulation [187], which would explain the commonly observed weight gain associated with thiazolidinedione therapy [34].

Some cancer types are known to express PLIN2 [90,91,92,93,95,96,99,101,105,106,107,108,160,161,162,163,164]. Consequently, PPARγ activators might heighten the expression of these PLIN isoforms, shielding FAs in the LDs more efficiently, thereby challenging the cancer cell’s ability to utilize FAs to meet its metabolic demands [157,188]. A more difficult access to FAs within the LDs could potentially suppress cancer cell proliferation. This hypothesis aligns with findings that TZDs have demonstrated a reduction in cancer cell proliferation [189] and that PPARγ activation is seen as a possible anticancer strategy [190,191,192].

TZDs are also known to have anti-inflammatory properties [193,194]. Whether the anti-inflammatory effects of thiazolidinediones could also be attributed to the inhibition of PLIN2 release and, hence, a consequential suppression of its function as an adipokine, is a possibility that merits further investigation.

### 5.2. AMPK Activators

Given AMPK function in promoting the CMA of PLIN2, PLIN3, and the increasing expression of PLIN5 [146], the stimulation of AMPK affords a potential route to altering the coordination of PLINs in the oversight of FA trafficking.

Pharmacologically, AMPK may be activated by drugs such as salicylic acid or by its precursor, acetylsalicylic acid [195] and metformin [177]. Therefore, it is expected that AMPK activators would ease the mobilization of FAs from LDs and prepare target cells for a more extensive use of FAs for mitochondrial β-oxidation. In 3T3-L1 preadipocytes, aspirin treatment was shown to inhibit adipocyte differentiation [196] and lipid accumulation [197]. In animal models of obesity, treatment with a low dose of aspirin resulted in a significant reduction of body weight, visceral fat mass and serum total cholesterols, and serum and adipose tissue TAGs [198]. In humans, the influence of aspirin on obesity has not yet been systematically studied but, in healthy volunteers, a low dose of aspirin was shown to increase β-oxidation [199].

Metformin was also shown to reduce LD accumulation in human chorionic villous mesenchymal stem cells [200], an observation consistent with what was expected from the effects of an AMPK activator. However, the inconsistency of the effects of metformin in human subjects did not support the approval for its clinical use for weight loss in non-diabetic patients [201]. The inconsistency observed could potentially be attributed to the mechanism underlying the activation of AMPK [201]. Metformin activates AMPK indirectly, inhibiting the respiratory chain complex I [202], ATP production and, consequently, causing a putative increase in the AMP/ATP ratio needed for AMPK activation. Weight loss caused by AMPK activation would also require FA β-oxidation, which will be limited in cases where respiratory chain complex I is inhibited.

Adiponectin is a glycoprotein primarily produced in adipocytes [203]. The expression of adiponectin and its plasma levels are influenced by circadian rhythms and by the activity of several hormones and transcription factors [204]. PPARγ is the major positive regulator of adiponectin gene expression [205], which provides a link between the availability of FAs for β-oxidation. The effects of adiponectin are mediated by adiponectin receptors, AdipoR1, and AdipoR2 [206]. Adiponectin receptors are membrane receptors with seven transmembrane domains like the G-protein coupled receptors [207]. However, they have an atypical structure and function, acting as catalytic receptors [208]. AMPK is one of the targets of adiponectin receptors [209]. Therefore, activation of adiponectin receptors by endogenous adiponectin or by adiponectin receptor agonists [210] may activate AMPK and cause the pattern of PLIN alterations expected from a direct AMPK activation: facilitating FA mobilization as a result of CMA of PLIN2 and PLIN3 [73] and promoting FA β-oxidation, as a result of the increase in PLIN5 expression [32]. Adiponectin also increases HDL (high-density lipoprotein) levels and its function [211] and the expression of apolipoprotein A-I (Apo A-I), the main apolipoprotein of HDL [212]. Single nucleotide polymorphisms in the PLIN1 and PLIN2 genes have been associated with changes in Apo A-I levels (PLIN1 in boys and PLIN2 in girls) and HDL-cholesterol levels (PLIN1 in girls), which was seen as a putative risk factor for obesity and cardiovascular disease depending on sex across life [213]. The influence of adiponectin in obesity and in cancer is widely accepted [214,215,216,217,218,219,220]. The role of adiponectin in preventing and protecting against the development of multiple disorders related to obesity, especially in metabolic syndromes, diabetes mellitus, cardiovascular diseases [221], inflammation [222], and cancers [217,223] has been extensively documented and may have a contribution from an effect on HDL levels, since it has been demonstrated that HDL can potentially reduce the risk and progression of cancer [213].

### 5.3. SIRT1 Activators

SIRT1 is a post-translational regulator that affects multiple biological processes according to the cell energy status [224], and its activity is induced by PLIN5 [225]. Therefore, SIRT1 activators may interfere with PLINs pathways by reinforcing the role of PLIN5 in promoting β-oxidation [149].

Resveratrol is one of the first SIRT1 activators studied, but the list of SIRT activators is being extended with the inclusion of other activators of natural (quercetin, sulforaphane) and synthetic origin (selisistat, SRT2104) [175,176]. Sulforaphane has been reported to decrease body weight and adipose tissue increases induced by HFD in mice, by reducing the number and size of LDs and the expression of PLIN2 and PLIN5 in 3T3-L1 cells [226]. The activity of SIRT1 activators in obesity and in related metabolic syndromes is being studied and some works revealed improvements in metabolic markers and mitochondrial function [227], which may indicate the involvement of SIRT1 activation in the effects of PLIN5 in preserving mitochondrial β-oxidation. Several studies have also demonstrated the potential effect of resveratrol in chemoprevention in some cancers [228]. Although several mechanisms have been proposed to explain the anti-cancer properties of resveratrol, the activation of PLIN5/SIRT1 pathway represents one likely explanation.

### 5.4. Modulators of cAMP/PKA Pathway

The cAMP/PKA pathway is a complex pathway that presents several possibilities of pharmacological intervention. One of the most obvious and extensively explored is the intervention at the membrane G-protein coupled receptors that lead to adenylyl cyclase activation [229]. The β-adrenoceptors (β-AR) are one of the most studied receptors [230].

β-AR activation promotes the lipolysis of stored triglyceride in both white and brown adipocytes [231]. The mechanism involved is PKA-induced PLIN1 phosphorylation and the consequent promotion of lipolysis [39]. However, this effect may not culminate in overall weight loss since β-AR activation also increases protein synthesis [232]. The anabolic effect explains the off-label use of β-AR agonists in animal production to increase growth and carcass protein composition [233]. It is also one of the reasons why β-AR agonists are included in the Prohibited List issued by the World Anti-Doping Agency [234,235].

In cancer, activation of the PKA pathway may also provide the energy to fuel the higher anabolic activity associated with these cells. This assumption is supported by the observation that clenbuterol, a long-acting β-AR agonist, was shown to induce cell proliferation [236] and that β-AR activation caused an increase in LD number and lipid content [170] in breast cancer cell lines. The putative clinical relevance of a pro-tumorigenic effect of β-AR activation is further supported by reports showing that the chronic use of β-AR antagonists decreases the incidence or mortality of many cancer types [237,238,239,240,241,242,243].

### 5.5. Other Drugs

Knowing the relevance of PLINs in controlling FA distribution and use, its putative involvement may be considered when trying to explain the effects caused by several drugs on body weight. For instance, the chronic use of antipsychotic drugs, such as olanzapine, clozapine, and chlorpromazine, causes an increase in body weight and fat mass [244,245]. These effects have been ascribed to increased expression of PLIN1 in human adipocytes [245], PLIN2, and PLIN4 [244].

The opposite may also occur. The following clinically approved drugs were described to decrease lipid accumulation or cause weight loss as an adverse drug reaction by a mechanism that involves a reduction of PLIN1 expression or an increase PLIN1 degradation: adapalene, used for topical treatment of mild to moderate acne [246]; artesunate, an antimalarial drug [247]; menadione, a synthetic form of vitamin K [248]; tetrandrine, an alkaloid with anti-inflammatory and anti-cancerous activities [249]; and nelfinavir, an antiviral agent [250].

Other drugs may cause a reaction pattern more complex than an increase/decrease of body weight. For instance, vitamin D [251] or dihydroartemisinin, an antimalarial drug, may reduce PLIN2 expression and LD formation [252].

## 6. Conclusions and Future Directions

This review summarizes the role of LDs and PLINs in the management of lipids in our body. The coordinated activity of PLINs promotes a healthy balance between storage, when supply exceeds demand, and release, when demand increases. However, this coordination is very fragile and can be easily disrupted.

Alterations in the balance of PLINs that cause an excessive retention of lipids in the LD may promote WAT expansion and obesity. This alteration may favor LD expansion in cancer tissues. Pharmacological interventions to prevent this type of imbalance could be applicable to the treatment of obesity-related diseases and cancer. The challenge to develop drugs that target the activity of PLINs may be facilitated by the knowledge that some drugs already used in clinical practice are known to alter body weight by altering PLINs activity. The present review is intended to provide new insights into the coordination of PLINs activity to foster the development of PLINs pharmacology, particularly with new classes of drugs that are able to directly target each PLIN.
